# The time is now: role of pragmatic clinical trials in guiding response to global pandemics

**DOI:** 10.1186/s13063-021-05165-0

**Published:** 2021-03-24

**Authors:** Aws Almufleh, Jacob Joseph

**Affiliations:** 1 Division of Cardiovascular Medicine, Brigham and Women’s Hospital, Harvard Medical School, Boston, MA USA; 2 Department of Medicine, Division of Cardiology, Queen’s University, Kingston, Ontario K7L 2V7 Canada; 3 Cardiology Section, VA Boston, Healthcare System, 1400 VFW Parkway, West Roxbury, MA USA; 4 Clinical Research Partnerships and Innovations, Massachusetts Veterans Epidemiology Research & Innovation Center (MAVERIC), Boston, USA

**Keywords:** Pragmatic clinical trials, COVID-19 pandemic, Electronic health records-embedded research, Adaptive trials design

## Abstract

Along with its heavy toll of morbidity and mortality, the coronavirus disease 2019 (COVID-19) pandemic exposed several limitations of the current global research response. The slow and inefficient process of carrying out traditional randomized clinical trials led regulatory authorities to hastily approve treatments and tests without sufficient evidence of safety and efficacy.

We here outline issues with the current research platform, summarize shortcomings of traditional randomized clinical trials particularly apparent at the time of pandemics, and highlight the advantages of pragmatic clinical trials as an alternative to rapidly generate the needed clinical evidence. We further discuss barriers and challenges to pragmatic clinical trials implementation and explore opportunities for research institutions and regulatory authorities to facilitate widespread adoption of this vital research tool.

As a subsequent wave of COVID-19, and/or another epidemic, are all but inevitable in our lifetime, we must ensure that our research infrastructure is conducive to carrying out pragmatic clinical trials to expeditiously generate the needed evidence and blunt the epidemic’s toll on human lives and livelihoods.

“There is a tide in the affairs of men, Which, taken at the flood, leads on to fortune” (Shakespeare, Julius Caesar).

## Background

In response to the escalating coronavirus disease 2019 (COVID-19) pandemic, the Food and Drug Administration (FDA) granted emergency use authorization for hydroxychloroquine to treat COVID-19 pneumonia in spite of questions about efficacy and safety [1–3]. Understandably, this was prompted by estimates of what was thought to be unacceptable delays in obtaining evidence from clinical trials. In this commentary, we propose that, by optimizing the current research ecosystem and accelerating regulatory approval for pragmatic (practical) clinical trials (PCTs), timely evidence generation can be accomplished to better guide drug approvals particularly at the times of pandemics. PCTs are defined as patient-centered, outcome-based trials examining the comparative benefits and risks of therapeutic interventions to inform clinical and/or policy decision-making [4]. These trials often employ electronic health records (EHR)-embedded research designs to rapidly test important clinical questions. PCTs are more feasible nowadays given the proliferation of collaborative research networks and integrated health systems (encompassing hospitals, clinics, and ambulatory services with unified EHR and central data warehouses) [5].

We herein summarize issues with the current research platforms, offer views on the advantages of PCTs, explore barriers to their widespread adoption, and list recommendations for successful implementation during the COVID-19 period and in the post-pandemic era.

### Challenges with the current clinical trial structure

Traditional randomized clinical trials (RCTs) are considered the highest level of evidence hierarchy. However, it has become increasingly apparent that RCTs are excessively burdensome to the clinical enterprise, and consist predominantly of narrowly focused RCTs with non-representative samples that generate “evidence” less applicable to real-world clinical settings [6]. This was best appreciated during COVID-19 pandemic. In a systematic review of 75 COVID registered clinical studies, investigators found that 80% of studies had a sample size less than 300 patients, and few studies included critically ill patients or used mortality as a primary outcome in their study design. Such methodological limitations will likely hinder clinical applicability of most of these traditional RCTs [7]. Another impediment to implementing traditional RCTs is the inefficient protocols requiring repeated research-specific in-person encounters for screening, consenting, study enrollment, and follow-up. The tribulation of these extraneous procedures has become particularly apparent during the pandemic when patients are reluctant to seek face-to-face care for medical reasons, let alone for the purpose of clinical research. Herein lies the value of PCTs; where patients’ screening and follow-up are embedded within the standard routine clinical care—thus addressing the unmet clinical needs without excess risk to patients/research participants.

### Advantages of pragmatic clinical trials

By far, the leading obstacle to performing traditional RCTs is the costly procedures required for screening patients, enrollment, collecting data, and event capture during follow-up [8]. PCTs, in contrast, utilize EHR-based strategies for automated screening and randomization, streamline consent processes, and incorporate follow-up assessments into routine clinic visits. By leveraging machine learning and advanced language processing tools, endpoint ascertainment can be achieved through automated periodic EHR review which facilitates interim analysis and safety monitoring [9]. In EHRs which lack the advanced language processing capabilities, more readily obtainable surrogate outcomes can be employed, e.g., drug discontinuation rates, patient hospitalization, and emergency visits. End-point confirmation could also be achieved through access to vital statistics from local/regional governmental authorities. Additionally, ease of data capture makes PCTs better suited to adaptive trial design that allows modification of tested interventions, sample size calculation, and expanding inclusion criteria to include a more representative patient sample. Although PCTs are often criticized for including heterogenous study populations and their vulnerability to unmasking and protocol deviation, testing in a real-world setting leads to more generalizable results which can promptly inform clinical practice. PCT methodology of broad inclusion criteria will capture most strata and severity levels of the disease and yield evidence that can be customized to patient groups frequently under-represented in RCTs (e.g., women, ethnic minorities, and critically ill patients).

Furthermore, the reduced per-patient cost allows large sample sizes in PCTs with advantages in adjusting for confounding which mitigates most protocol deviations that do not infrequently occur in trials. Additionally, unlike traditional RCTs, PCTs with large sample sizes are often powered to assess multiple interventions and outcomes including patient-centered outcomes (e.g., quality of life), health service delivery metrics (efficiency and cost-effectiveness), and traditional clinical outcomes. Importantly, once the infrastructure for a successful PCT has been established, it can be deployed for future studies, which facilitates time-sensitive response, particularly during global pandemics. This need was previously recognized in the Institute of Medicine (IOM) report from 2010 that suggested streamlining the clinical trials infrastructure so that those investigating new research questions could quickly draw on resources already in place instead of reinventing the wheel for every trial [8].

### Barriers to pragmatic clinical trials implementation

The lack of mainstream adoption of PCTs stems from several barriers. First, despite expedited and streamlined subject screening, randomization, and treatment allocation processes, the current regulatory and approval process is often protracted and resource-intensive [4]. Multicenter multinational trials are even more cumbersome to approve and require fulfilling varying local and national auditing/regulatory processes in different sites [8].

Furthermore, despite encouraging guidelines from the Department of Health and Human Services to streamline regulatory approval of multicenter trials, labeled as avoiding “battle of the forms” initiative, following these recommendations across varying regulatory boards remains the exception, not the rule [10]. Another barrier is cost, which though substantially less than traditional RCTs, could still be an obstacle when resources are shunted to frontline clinical activities and in areas where research funding is not a recognized priority of public health spending. Though some barriers could theoretically be mitigated by leveraging advanced features of high functionality EHR systems, this may not be always attainable. For example, across many institutions, EHRs are fragmented, difficult to interrogate, and are not prepared to serve the fundamental needs of PCTs (including automatic participant screening, streamlined enrollment and follow-up, and outcomes adjudication). Furthermore, the widespread heterogeneity in EHR systems can impede streamlined data sharing which is pivotal for national and multinational collaborations.

Importantly, some obstacles to the adoption of PCTs are largely based on misconceptions. Contrary to the pervasive belief that patients are reluctant to participate in trials during pandemics, a large survey has shown overwhelming acceptance and enthusiasm from the public [11]. The observed low enrollment rate with traditional RCTs is likely related to a lengthy consenting process and burdensome study-specific requirements. Furthermore, the concern about inaccurate EHR-derived subject screening or event ascertainment is misplaced; for example, a recent report showed that a well-designed EHR-based screening algorithm had an overall accuracy of 96% in curating a complex condition such as heart failure with preserved ejection fraction [12]. Additionally, the difficulty of individual-level patient randomization in PCTs can be mitigated by cluster-randomized trial design with cross-over phases to combine sufficiently rigorous methodology and ease of implementation.

### Opportunities and resources

In order to overcome the above obstacles, a concerted public-private collaboration is needed. This must involve all key stakeholders including governments, regulatory authorities, academia, pharmaceutical industry, and regional/global organizations, such as the World Health Organization (WHO). Relevant to the *journal’s* readers, we here summarize the potential roles and opportunities of two stakeholders in ensuring success and widespread implementation of PCTs; academic/research institutions and the regulatory authorities.

#### Academic/research institutions

Academic institutions are poised to play a key role in research response to global pandemics. Their role in patient care and access to clinical databases position them to identify the most pressing clinical questions and leverage their established research infrastructure to produce the needed evidence. However, most research output generated during the COVID-19 pandemic represents single-center experiences rather than the needed impactful multicenter, multinational work. Needless to say, siloed scholarly experts must join forces to focus their efforts, avoid redundancy, and address the more pressing clinical questions. An encouraging, though uncommon, display of this needed collaboration is best exemplified in the multinational Remap-Cap pneumonia treatment trial (NCT02735707), randomizing over 600 COVID-19 patients in 180 sites across 14 countries [9]. Their use of adaptive trial design with rapid enrollment, patient representation/engagement, and prompt inclusion of COVID-19 treatment arm even though the trial had already been running is commendable and reflects the essence of PCT *in action*. By running an EHR-embedded study across many collaborating sites, the study investigators managed to address several important clinical questions at a fraction of the time and operating cost of similarly sized traditional RCTs.

The goal of widespread academic collaboration to facilitate multicenter research endeavors is certainly ambitious and, to the skeptics, may seem far-fetched. However, it is important to recognize that several global and national research networks have already been formed in response to prior public health epidemics including the International Severe Acute and Emerging Infectious Diseases Consortium among several others.

Rather than aiming to construct new collaborative networks, building on established partnerships particularly those with expertise in PCT design and implementation should be pursued. A notable example of these networks includes the largest EHR-linked health system in the USA, the Veterans Affairs Healthcare System (VA), which is currently conducting several PCTs with remarkable success (examples include ClinicalTrials.gov NCT02185417 and NCT04262206). Another network is the National Patient-Centered Clinical Trials Network which is a National Institute of Health-funded organization linking multiple integrated health systems and research networks into a national consortium for patient-centered outcomes research. The logistics of transitioning available networks to adapt infectious disease/public health-centered focus when needed remains to be elucidated and is beyond the scope of this article.

#### Regulatory authorities

At the time of pandemics; when rapid implementation of PCTs can *effectively alter the global burden of disease*, expedited regulatory approval is simply paramount. Notably, COVID-19 pandemic led several regulatory authorities to “over-correct” for the built-in delay in the RCT platform by granting emergency use authorizations to tests and treatments before efficacy was established in clinical trials [1]. One can argue that tailoring the upstream approval process to facilitate PCT implementation would promptly generate the required evidence and potentially obviate the need for some emergency use authorizations.

Two areas have been widely recognized as bottlenecks for the approval of clinical trials; consenting process, and monitoring/documentation requirements particularly in multicenter and multinational trials.

#### Consent requirements

The current consenting process is well-known to be excessively lengthy and inefficient. During the pandemic, the embedded mechanisms enabling remote contact with patients using EHR at most established PCT infrastructures could be conducive to rapid trial enrollment if virtual consenting is widely endorsed. For the acutely ill patients who are unable to provide informed consent, remote consenting processes can be employed to reach caregivers and legal decision makers who are often not allowed to enter medical facilities during the pandemic.

#### Monitoring/documentations

Since 2010, the FDA-Duke University collaboration project, titled the Clinical Trials Transformation Initiative, noted that most of the costs incurred in clinical trials are associated with human time and effort, mostly to ensure adequacy of completing required forms and repetitive site audits [8].

Embracing risk-based central monitoring with automated data feeding will not only save resources but allow correcting deviations during trials as opposed to the predominant method of retrospective auditing. Furthermore, the use of information technology to streamline and facilitate regulatory requirements should be universally adopted. Finally, the burden of documentation should be commensurate to their relevance to patient safety and to establishing treatment efficacy rather than mere affirmation of exhaustive document completion.

## Conclusion

It has been over 50 years since epidemiologists Daniel Schwartz and Joseph Lellouch described the emerging role of pragmatic clinical trials and their value in generating evidence readily applicable to real-world clinical settings [13]. Perhaps a “silver lining” of the COVID-19 pandemic is that it magnified the need for such trials and will push the scientific community closer to widespread adoption of PCTs. The rapid spread of COVID-19 is a reminder that we need a commensurately swift response in generating high-quality clinical evidence with rapid implementation. Pragmatic clinical trials are well-positioned to be at the forefront of this coordinated response.

## Data Availability

Not applicable

## Abbreviations

COVID-19: Coronavirus disease 2019
EHR: Electronic health records
FDA: Food and Drug Administration
IOM: Institute of Medicine
PCTs: Pragmatic clinical trials
RCT: Randomized clinical trials
VA: Veterans Affairs Healthcare System
WHO: World Health Organization

## References

[1] Biomedical Advanced Research and Development Authority (2020). Request for Emergency Use Authorization For Use of Chloroquine Phosphate or Hydroxychloroquine Sulfate Supplied From the Strategic National Stockpile for Treatment of 2019 Coronavirus Disease.

[2] Pascarella G, Strumia A, Piliego C, Bruno F, Del Buono R, Costa F, et al. COVID-19 diagnosis and management: a comprehensive review. J Intern Med. 2020;288(2):192–206. 10.1111/joim.13091. 10.1111/joim.13091 PMC7267177 32348588

[3] Geleris J, Sun Y, Platt J, Zucker J, Baldwin M, Hripcsak G (2020). Observational study of hydroxychloroquine in hospitalized patients with Covid-19. N Engl J Med.

[4] Califf RM, Sugarman J (2015). Exploring the ethical and regulatory issues in pragmatic clinical trials. Clin Trials..

[5] Bertagnolli MM, Anderson B, Quina A, Piantadosi S. The electronic health record as a clinical trials tool: opportunities and challenges. Clin Trials. 2020;17(3):237–42. 10.1177/1740774520913819. 10.1177/1740774520913819 32266833

[6] Yusuf S, Collins R, Peto R (1984). Why do we need some large, simple randomized trials?. Stat Med.

[7] Zhu RF, Gao YL, Robert SH, Gao JP, Yang SG, Zhu CT. Systematic review of the registered clinical trials for coronavirus disease 2019 (COVID-19). J Transl Med. 2020;18(1):274. 10.1186/s12967-020-02442-5. 10.1186/s12967-020-02442-5 PMC7338108 32631442

[8] Transforming Clinical Research in the United States (2010). Challenges and opportunities: workshop summary.

[9] Angus DC, Berry S, Lewis RJ, Al-Beidh F, Arabi Y. Van Bentum-Puijk W, et al. The randomized embedded multifactorial adaptive platform for community-acquired pneumonia (REMAP-CAP) study: rationale and design. Ann Am Thorac Soc. 2020;17(7):879–91. 10.1513/AnnalsATS.202003-192SD. 10.1513/AnnalsATS.202003-192SD PMC7328186 32267771

[10] The U.S. Department of Health and Human Services (“HHS”) Secretary’s Advisory Committee on Human Research Protections. Transition Provisions and Informed Consent Req. https://www.hhs.gov/ohrp/sachrp-committee/recommendations/attachment-e-november-13-2018/index.html.

[11] Gobat N, Butler CC, Mollison J, Francis NA, Gal M, Harris V (2019). What the public think about participation in medical research during an influenza pandemic: an international cross-sectional survey. Public Health.

[12] Patel YR, Robbins JM, Kurgansky KE, Imran T, Orkaby AR, McLean RR (2018). Development and validation of a heart failure with preserved ejection fraction cohort using electronic medical records. BMC Cardiovasc Disord.

[13] Schwartz D, Lellouch J (1967). Explanatory and pragmatic attitudes in therapeutical trials. J Chronic Dis.

